# Lectures on Physiology, Zoology and the Natural History of Man

**Published:** 1832

**Authors:** 


### REVIEWS

### AND BIBLIOGRAPHICAL NOTICES.

---

Art. V. *Lectures on Physiology, Zoology, and the Natural History of Man*, delivered at the Royal College of Surgeons— By W. Lawrence, F. R. S., Professor of Anatomy and Surgery to the College, Assistant Surgeon to St. Bartholomew's Hospital, Surgeon to Bridewell and Bethlehem Hospitals, and to the London Infirmary for Diseases of the Eye.

We gave in our last number some account of Lawrence's Lectures, confining our remarks to that part of the work which treated of the *Specific Characters of man.* In the present number, we design to continue our review, and present some of the author's views on the *varieties of the human species.*

Are all mankind of one species? This question has been connected with so many associations of religion, sympathy, and self interest, that its investigation is beset with difficulties calculated to mislead the mind, and obstruct the course of free investigation. Records, which are guarded with sleepless jealousy by the popular religion of the whole civilized world, appear very clearly to deduce the different families of mankind from a common parent. Philanthropy, in view of the fearful ravages which war and oppression have produced, have endeavored to enlist the sympathies of a common nature in opposition to the destructive influences of self interest and ambition. Scholastic Philosophy, with her ponderous weapons of unintelligible logic, and *a priori* reasoning, has not been wanting in her efforts; nor, if the object of reasoning be conviction without illumination, have her labors been in vain.

The period, however, has long since passed by when truth could successfully appeal to the tribunal of bigotry or scholastic learning. Her proper weapons are investigation and observation, and with these, she should be always ready to enter the lists without a fear of the consequences.

The differences, in the physiological structure—in the form, size, colour, &c. of the different races of mankind, and in their moral, social and intellectual condition, are so striking as very obviously to lead superficial observes to the belief of a difference in the species; but if the points of difference are great, those of resemblance are not less obvious, and are more fundamental. Different persons, consequently, from diversity of views or other motives, have taken opposite sides of the question and defended their opinions with great plausibility. Under these circumstances, there is but one common and satisfactory ground upon which all enquirers can meet, and that is the ground of natural history and physiology. We can hope to ascertain and establish the true state of the case, only by comparing the differences among the different races of mankind, with those which we observe among animals; by ascertaining the influence of natural and moral causes upon both, and applying to the information thus derived from observation and analogy, the aid of physiology and comparative anatomy. If in the pursuit of this method of investigation, we arrive at conclusions which will confirm the sanctions of religion, and give full play to the benevolent and sympathetic principles of our nature, the friends of truth will have double cause of congratulation in the result of their labors.

Before entering upon this investigation, it is necessary to determine the precise import of the terms *species* and *variety.*

It may be stated in the abstract, that all animals which differ in such points only as might arise in the natural course of degeneration, that is, from recognised causes of variation, belong to the same species; while those differences which cannot be accounted for on this supposition, must lead us to class the animals which exhibit them in different species. But the chief difficulty is, to point out the characters by which, in actual practice, mere varieties may be distinguished from genuine specific differences.

The different species of animals are distinguished by fixed and definite external forms which, in general, are perpetuated with little or no variation from generation to generation. These characters, however, may be modified by a variety of circumstances. Climate produces some slight influence, and variations in the quantity and quality of the food still more. But as animals, when left to themselves, generally select as their place of abode, the situation and climate adapted to their organization and wants, the differences produced by these circumstances among wild animals are, for the most part, very slight.

Domestication produces the greatest changes, and the extent of its influence depends upon the more or less perfect subjection of the animal to the will or caprice of his master, and especially when man regulates their sexual intercourse, by selecting individuals to breed from, the most astonishing changes in form and qualities have been produced. The herbiverous domestic animals, the companions of man in almost every climate, exhibit changes which are indeed very striking but chiefly superficial. The greatest influence, however, has been produced upon the dog, in which such changes have been produced that the different varieties vary more widely than any wild species of the same genera. Still, however, in all these cases, the connection of the bones remains the same, and the form of the teeth is never altered.

The criterion of breeding was at one time, through the influence of Hunter and Buffon, very generally received as an evidence of the identity of species. The aversion to an union between the different species of animals, and the general sterility of hybrid animals led these naturalists to adopt the principle that when a union resulted in a prolific offspring the differences were the result of adventitious circumstances. The admission of this principle would at once settle the question as to the human race, but the criterion is liable to insuperable objections.

This rule, however, involves a *petitio principii*, in assuming that animals of distinct species never produce together a prolific offspring. Generally, indeed, hybrid animals, or the offspring of any two species, are incapable of generation; and this is a powerful additional provision for preserving uniformity of species. There are, however, instances, both among the mammalia and birds, of individuals belonging to species universally held to be distinct, uniting and producing young, which were again prolific. That the mule can engender with the mare, and that the she mule can conceive, was known to Aristotle. The circumstance is said to occur most frequently in warm countries; but it has taken place in Scotland. Buffon states that the offspring of the he-goat and ewe possesses perfect powers of reproduction. We might expect these animals, with the addition also of the chamois, (antelope rupicapra) to copulate together easily, because they are nearly of the same size, very similar in internal structure, accustomed to artificial domestic life, and to the society of each other from birth upwards. There is a similar facility in some birds belonging to the genera fringilla, anas, and phasianus, where such unions are often fruitful and produce prolific offspring. The cock and hen canary-birds produce with the hen and cock siskin and goldfinch; the hen canary produces with the cock chaffinch, bullfinch, yellow-hammer, and sparrow. The progeny in all these cases is prolific, and breeds not only with both the species from which they spring, but likewise with each other. The common cock and the hen partridge, as well as the cock and the guinea hen,|| the pheasant and the hen, can produce together.

The anser cygnoides (Chinese goose) copulates readily in Russia with the common goose, and produces a hybrid but perfectly prolific offspring: the race soon returns to the characters of the common goose, unless crossed again with the Chinese species.

The different breeds of dogs, for example, are referred by some to different species; and they are indeed sufficiently marked by distinctive permanent characters to warrant the opinion, if the constancy of such characters were a sufficient proof of difference in species.—Others, again, refer them all to the shepherd's dog; and others include all the dogs, the wolf, fox, and jackall, in one species. The dog and bitch produce with the male and female wolf, and with the dog and bitch fox, and the offspring is prolific. Yet we cannot surely ascribe animals which are marked in their wild state by such strong characters, of bodily formation, disposition, and habits—as the wolf, fox, and jackall, to one and the same species, without overturning all the fundamental principles of zoology, however freely they may intermix, and however perfect the reproductive power may be in their offspring.

We may conclude, then, from a general review of the preceding facts, that nature has provided, by the insurmountable barriers of instinctive aversion, of sterility in the hybrid offspring, and in the allotment of species to different parts of the earth, against any corruption or change of species in wild animals. We must therefore admit, for all the species which we know at present, as sufficiently distinct and constant, a distinct origin and common date. On the other hand, the fruitful intermixture which art has accomplished, of some of these species, will not justify us in ascribing to them identity of race or origin, when we see them in the natural wild state distinguished by constant characters from the typeof the neighboring species, and always producing an offspring marked by these characters.

Since this criterion therefore is inadmissible, the author resorts to the mode recommended by Blumenbach, which is, to draw our notions of species in zoology from analogy and probability.

If we see two races of animals resembling each other in general, and differing only in certain respects, according with what we have observed in other instances, we refer them without hesitation to the same species, although the difference should be so considerable as to affect the whole external appearance. On the contrary, if the difference should be of a kind which has never arisen, within our experience of the animal kingdom, as a variety, we must pronounce them to belong to distinct species, even although there should be on the whole, a great general resemblance between the two. "I see," says this acute and judicious naturalist, "a remarkable difference between the Asiatic and African elephants, in the structure of the molar teeth.—Whether these inhabitants of such distant regions will ever be brought to copulate together, and whether this formation be universal, is uncertain; but it exists in all the specimens I have seen or heard of; and I know no example of molar teeth changed in such a manner by degeneration or the action of adventitious causes: therefore I conjecture from analogy, that these elephants are not mere varieties, but truly different species. On the other hand, I hold the ferret (mustela furo) to be only a variety of the pole-cat (m. putorius,) not so much because they produce together, but because it has red pupils: and the analogy of numerous other instances induces me to regard all the other mammalia which are destitute of the coloring pigment of the eye, as varieties degenerated from their original stocks."

In chapter II. Dr. L. after a general description of the skin, proceeds to give an account of the diversities of colour in the human race. These tints, as the author remarks, are innumerable, embracing every gradation of colour, from the clear snowy white of the European female to the jet black colour of the darkest African. Notwithstanding these tints are separated by insensible gradations the national varieties of colour may with sufficient accuracy be referred to five principal classes.

1. *White,* to which redness of the cheeks is almost wholly confined, being observed, at all events, very rarely in the other varieties. It is seen in all the European nations, excepting the Laplanders; in the western Asiatics, as the Turks, Georgians, Circassians, Mingrelians, Armenians, Persians, &c.; and in the northern Africans.

II. *Yellow* or *olive* (gilvus or buxeus, a middle tint between that of ripe wheat and boiled quince or dried lemon-peel) characterizes the

Mongolian tribes, usually called, together with the inhabitants of great part of Asia, Tartars (Tatars.)

III. *Red or copper color* (*bronze* Fr. an obscure orange or rusty iron color, not unlike the bark of the cinnamon-tree) prevails in various shades over nearly the whole continent of America, and is almost confined to that division of the globe.

IV. *Brown or tawny* (*basane* Fr. a middle tint between the color of fresh mahogany and of cloves or chesnuts.) It characterizes the Malays, and most of the inhabitants of the numerous islands scattered through the Pacific Ocean.

V. *Black*, in various shades from the sooty color or tawny black, to that of pitch or ebony, or jet-black. This prevails very extensively on the continent of Africa, characterizing all the Negro tribes. It is found also in the Negro-like natives of New Holland, Van Diemen's Land, Papua or New Guinea, the New Hebrides, and other islands of the South Sea; and is seen, mingled with the national color, in Brazil, California, and India. The New Caledonians constitute an insensible transition, with the chesnut-colored islanders of Tongataboo, and the dark New Hollanders, from the tawny or brown Otaheiteans to the Papuas or Negroes of New Guinea.

In describing these varieties, it is necessary to fix on the most strongly marked tints, between which there is every conceivable intermediate shade of color. The opposite extremes run into each other by the nicest and most delicate gradations; and it is the same in every other particular in which the various tribes of the human species differ. This forms no slight objection to the hypothesis of distinct species: for, on that supposition, we cannot define their number, nor draw out the boundaries that divide them: whereas, in animals most resembling each other, the different species are preserved pure and unmixed. Neither does the color, which I have described in general terms as belonging to any particular race, prevail so universally in all the individuals of that race as to constitute an invariable character, as we should expect if it arose from a cause so uniform as an original specific difference; its varieties, on the contrary, point out the action of other circumstances. Thus, although the red color is very prevalent on the American continent, travellers have observed fair tribes in several parts; as, Ulloa and Bouguer in Peru; Cook and Vancouver at Nootka Sound; Humboldt near the sources of the Orinoco; and Weld near the United States. The natives of New Zealand vary from a deepish black to an olive or yellowish tinge. In the Friendly Islands many of the women are as fair as those of Spain or Portugal; several of both sexes are of an olive color; and many of a deep brown.

Children produced from the union of two different races exhibit the middle tint between those of their parents. This law holds universally, climate not making the slightest difference.

The general law, that animals produce their like, is liable to occasional exceptions; and hence in each of the races, persons are occasionally produced with characters resembling those of the opposite races. Variations of colour analogous to these occur among animals, and if these varieties are encouraged and preserved from intermixture, they form the ground for a permanent diversity of breed; otherwise the disposition to change is exhausted in one generation. In considering this as an explanation of the mode in which varieties of colour occur in the human race, it is not necessary to the adoption of the hypothesis that among any of the races varieties should arise presenting the full contrast of the greatest diversity which occurs in the human family. The negro and the European are the extremes of a very long gradation, and between these are intermediate stages, presenting no greater differences than are every day met with among the individuals of every race.

*Chapter III.* is devoted to the *hair, beard, and color of the iris.* The hair being implanted in the skin, and deriving its growth from the cutaneous vessels, might be supposed to vary with the changing conditions of the skin. The substance of which it is composed is indeed very analogous to that of the cuticle, being, like it, destitute of vessels, nerves, sensibility, and every other evidence of vitality. As an evidence that this presumption coincides with the fact, it may be remarked that the four colored varieties of men, the texture of whose skin is much denser and thicker than that of the whites, have black, strong and coarse hair. The varieties of the human hair are arranged by the author under the following heads.

1. Brownish, deviating into yellow (flaxen) or red on one side, and black on the other; copious, soft, long, and forming more or less distinct ringlets or undulations. It is seen in the temperate climates of Europe, and its light shades formerly attracted particular notice in the ancient Germans. The thin-skinned Albino has the softest and most colorless hair: in the Germanic race it is also very soft and light-colored; and red hair is usually found in conjunction with a thin and soft skin. The Celtic and Sclavonic races, which make up the chief population of Europe, the eastern Asiatics, and northern Africans, have generally, with a rather thicker and darker skin, stronger, black, or dark brown, and more or less curling hair.

The lighter and darker kinds of hair will grow to very considerable lengths in Europeans, when not cut.

2. Black, strong, straight, and thin; in the Mongolian and American varieties. The greater part of the head is shaved by the Chinese; the portion of hair which they leave, often reaches the ground. The same remark holds good of the Americans.

3. Black, softer, dense, copious, and curled; in most of the South Sea Islanders.

4. Black and crisp, so as generally to be called woolly; common to all the Negro tribes. This is either formed into small and short masses, or it may admit of being combed to the length of three or four inches, still forming a kind of general woolly fleece.

This division, as a general one, is sufficiently accurate, although there are many exceptions to it. A similar diversity in regard to the color and texture of the hair, we find in the animal kingdom among those which have evidently descended from the same stock.

The different races of mankind exhibit considerable diversity in the beard, and the hair covering other parts of the body, as well as in that of the head. One of the most general characters of the dark colored races is a deficiency of the beard. And in connection with this circumstance we have a fact which goes far towards accounting for the diversities of the human race, viz; that all national peculiarities are cultivated as personal ornaments. In conformity with this principle almost all barbarians labor to eradicate the beard.

There is also a connexion between the color of the skin and the eyes, analogous to that pointed out between the skin and the hair. We see this in the proverbial connexion between the complexion and the eyes, as well as in the varying color of the hair and eyes in the different stages of human life. The different shades of the iris may all take place in different individuals of the same races, but they are also, sometimes characteristic of different races. Thus, blue eyes and yellow hair (cœrulei oculi rutilæ comæ) have characterized the German nations from the earliest history; and Linneus describes, in Sweden, the Gothlander with light hair and grayish blue eyes; the Fin with yellow hair and brown eyes: and the Laplander with dark hair and eyes. The iris is dark in all the colored tribes of men, as well as in the dark complexioned individuals of the white variety, and in the negro is sometimes so dark, that close inspection is necessary to distinguish it from the pupil.

Chapter IV.—*Treats of Differences of Features; Forms of the Skull; Teeth, &c.*—The five following varieties are established by Blumenbach, after a careful comparison of numerous drawings of individuals from different countries; in situations where they are attracted by commerce from all parts of the world, as at London and Amsterdam. The distribution of course, only refers to leading traits, between which there are innumerable varieties, running into each other by insensible gradations.

1. An oval and straight face, with the different parts moderately distinct from each other; high and expanded forehead; nose narrow, and slightly aquiline, or at least with the bridge somewhat convex; no prominence of the cheek bones; small mouth, with lips slightly turned out, particularly the lower one; a full and rounded chin. This is the kind of countenance which accords most with our ideas of beauty: it may be considered as a middle, departing into two extremes, exactly opposed to each other in most respects, yet agreeing in having a low and receding forehead. In one, the face is expanded laterally; in the other, it is lengthened forwards or downwards. Each of these includes two varieties, which are most readily distinguished by a profile view; one, in which the nose and other parts run together; and the other, in which they are more prominent and separate.

2. Broad and flattened face, with the parts slightly distinguished, and as it were running together: the space between the eyes flat and very broad, flat nose, rounded projecting cheeks: narrow and linear aperture of the eyelids extending towards the temples, (*yeux brides*, Fr.) the internal angle of the eye depressed towards the nose, and the superior eyelid continued at that part into the inferior by a rounded sweep: chin slightly prominent.

This is the face of Mongolian tribes; commonly called in English the Tartar face, from the confusion of the Tartars (Tatars) with the Mongols.

3. Face broad but not flat and depressed, with prominent cheek-bones; and the parts, when viewed in profile, as it were more deeply and distinctly carved out. Short forehead, eyes deeply seated, nose flattish, but prominent. Such is the countenance of most Americans.

4. Narrow face projecting towards its lower part; narrow, slanting, and arched forehead; eyes prominent (*a fleur de tete,*) a thick nose, confused on either side with the projecting cheeks (*nez epate;*)

the lips, particularly the upper one, very thick; the jaws prominent, and the chin retracted. This is the countenance of the negro—the Guinea face.

5. The face not so narrow as in the preceding, rather projecting downwards, with the different parts in a side view rising more freely and distinctly. The nose rather full and broad, and thicker towards its apex (bottle-nosed.) The mouth large. This is the face of the Malays, particularly of the South-Sea Islanders.

From the consideration of the countenance, we pass by an easy transition to the various formations of the skull. We are indebted to Blumenbach for the principal part of the information we possess upon this subject, who has devoted to it a great deal of labor, and has been enabled to illustrate it by a collection of the skulls of different nations from every part of the globe.

The differences between the proportions and the direction of the various parts of the head in different skulls, is so great, that it is difficult to reduce them to any one scale of measurement, or to compare them with each other at one view. The point of view which Blumenbach recommends, as best calculated to present, at one glance, the various differences which make up the national character of the cranium, is the following. The skulls are placed on a table, with the zygomas perpendicular, and viewed from behind; and we thus are enabled to perceive at once the most important points of the head; the direction of the jaws and cheek bones, the breadth or narrowness of the head, and the advancing or receding outline of the forehead. This method of considering the bony head B. calls the *norma verticalis.* This arrangement of the different races, drawn from the form of the skull, will coincide in many respects with that pointed out by the diversity of the features.

In the first, or white variety of man, to which Blumenbach has given the epithet *Caucasian*—including the ancient and modern inhabitants of Europe, the western Asiatics, or those on this side of the Caspian Sea, the rivers Ob and Ganges, and the northern Africans; in a word, nearly all the inhabitants of the world as known to the ancients,—the skull presents the finest intellectual organization; proportions indicating the greatest predominance of the rational faculties over the instruments of sense and of the common animal wants. The upper and front parts of the skull are more developed than in any other variety; and their ample swell completely hides the face, when we survey the head according to the *norma verticalis*. The facial line must, therefore, be nearly vertical; and the facial angle nearly a right angle. The face is comparatively small, and its outlines rounded, without any thing harsh or unpleasantly prominent. The cheek bones are small, and do not stand out, but descend in a nearly straight line from the external angular process of the frontal bone. The alveolar margin of the jaws is rounded; and the front teeth are perpendicular in both. The chin is full and prominent.

The characters above described belong to the following people, whether ancient or modern; viz. the Syrians and Assyrians, Chaldeans, Medes, Persians, Jews, Egyptians, Georgians, Circassians, Mingrelians, Armenians, Turks, Arabs, Afghans, Hindoos of high caste, Gipsies, Tartars, Moors, and Berbers in Africa, Guanches in the Canary Islands, Greeks, Romans, and all the Europeans except the Laplanders. The enumeration includes all the human races in which the intellectual endowments of man have shone forth in the greatest native vigor, have received the highest cultivation, and have produced the richest and most abundant fruits in philosophy, science and art, in religion and morals, in poetry, eloquence, and the fine arts, in civilization and government—in all that can dignify and ennoble the species. We cannot, therefore, wonder that they should in all cases have not merely vanquished, but held in permanent subjection, all the other races.

A very great difference of opinion has prevailed upon the question, whether the ancient Egyptians belonged to the Caucasian, or to the Ethiopian variety, and the opinion has very extensively prevailed, that they belonged to the latter. It is, however, at present the opinion of Cuvier, Blumenbach, and of all anatomists and naturalists who have had opportunity of examining the crania of Egyptian mummies, that they belonged to the Caucasian variety. Occasional specimens are met with bearing traces of the Negro conformation, but the greater number by far, are evidently not of that race.

The four following varieties exhibit, in comparison with the former, a less perfect development of the upper and anterior parts of the cranium, and consequently a comparatively inferior organization of the intellectual faculties. It is to be recollected, however, that this remark is to be understood in a very general sense. In forming a classification of this kind, the extreme points of difference are sought for, to render the contrast more complete, and consequently, we will expect to find in each of the classes individuals, whose organization and mental faculties partake of the characters of the opposite race.

Every white man has not the mental endowments of a Newton, nor do we expect to find the signs of intellectual inferiority in the forehead of every Negro.

The second or Mongolian variety includes those Asiatics which do not come under the first division, and the inhabitants of the northern parts of America and Europe. The forehead is low and slanting, and the head altogether of a square form. The cheek bones stand out widely on either side. The glabella and ossa nasi, which are flat and very small, are placed nearly on the same plane with the malar bones. There are scarcely any superciliary ridges. The entrance of the nose is narrow; the malar fossa forms but a slight excavation. The alveolar edge of the jaws is obtusely arched in front; the chin rather prominent. This formation is most strikingly exhibited in the Mongolian tribes, which are widely scattered over the continent of Asia, and which have generally, but erroneously been included, with others of different origin and formation, under the name of Tartars (Tatars;) whereas the last-mentioned tribes, properly so called, belong to the first division of the human race. The Calmuck and other Mongolian nations which overran the Saracen empire under Zenghis Khan, in the thirteenth century, and had entered Europe, are described in the *Historia Major* of Matthew Paris, under the name of *Tartars;* whereas that appellation, or rather Tatars, properly belongs to the western Asiatics, who had been vanquished by the Mongols. The error, however, arising from this source has been propagated down to the present day; so that in the works of the most approved naturalists, as Buffon and Erxleben, we find the characters of the Mongolian race ascribed to what they call the Tartars. The mistake has not been detected, even by the most celebrated and classical modern historians: For Dr. Robertson speaks of Zenghis as the Emperor of the Tartars.

The third or Ethiopian variety comprehends all the Africans which are not included within the first or Caucasian division; all of whom partake, more or less, of the well known Negro form.

The front of the head, including the forehead and face, is compressed laterally, and considerably elongated towards the front; hence the length of the whole skull, from the teeth to the occiput, is considerable. It forms, in this respect, the strongest contrast to that globular shape which some of the Caucasian races present, and which is very remarkable in the Turk.

The capacity of the cranium is reduced, particularly in its front part, where it appears as if the forehead had been sliced off. The face, on the contrary, is enlarged.

"I measured," says Soemmering, "several Negro, and nearly all my European crania, in order to compare the capacity of the respective cerebral cavities. I found in the former, 1st, That the measure taken by carrying a string from the root of the nose, along the middle of the forehead and the sagittal suture, to the posterial edge of the foramen ovale, the length of the face being equal, was much shorter. 2dly, That the horizontal circumference, measured by a string carried round the head above the eyebrows and the superior edge of the temporal bone, was much less. 3dly, That neither the long diameter from the forehead to the occiput, nor any transverse diameter between the parietal or the temporal bones, is equal to the corresponding one in the European."

The characters of the Ethiopian variety, as observed in the genuine Negro tribes, may be thus summed up; 1. Narrow and depressed forehead; the entire cranium contracted anteriorly; the cavity less, both in its circumference and transverse measurements. 2. Occipital foramen and condyles placed farther back. 3. Large space for the temporal muscles. 4. Great development of the face. 5. Prominence of the jaws altogether, and particularly of their alveolar margins and teeth; consequent obliquity of the facial line. 6. Superior incisors slanting. 7. Chin receding. 8. Very large and strong zygomatic arch projecting towards the front. 9. Large nasal cavity. 10. Small and flattened ossa nasi, sometimes consolidated, and running into a point above.

In the two following varieties, the figure of the skull is not so strongly characterized as in the three which have been already considered. They form, indeed, two intermediate gradations between the European and Mongolian on one side, and the African on the other.

The fourth, or American, variety includes all the Americans, except the inhabitants of the northern parts of the continent, which I have placed in the Mongolian division.

In this variety the cheeks are broad, but the malar bones are more rounded and arched than in the Mongolian; and not expanded to such an extent on either side, nor possessing such an angular form. The forehead is small and low, the orbits deep; and the nasal cavity, in many cases at least, very large. The entire bony apparatus of the face is in general much developed.

The fifth or Malay variety, including the inhabitants of numerous Asiatic Islands, and those of the Great Pacific Ocean, constitutes an intermediate link between the European and Negro. The cranium is moderately narrowed and slanting at its anterior and upper part; the face large, and all its parts fully developed; the jaws more or less prominent.

It must be confessed, that the numerous tribes included within the boundaries of this variety differ considerably from each other; and consequently, that the whole cannot fall within one clearly marked character. The Papua race are described as having all the appearance of Negroes; I have seen no skull, nor any representation of one belonging to a native of New Guinea. The New Hollanders certainly partake of the Negro form, yet are still easily distinguishable from

African Negroes. In the two heads engraved by Blumenbach, the forehead rather slants above the eyes, but the head rises to a considerable height at the coronal suture. The nose is not so flat, nor the zygoma so prominent, as in the African. The alveolar edge of the upper jaw projects in front; the chin is not cut off, as in the Negro. The crania of New Hollanders which I have seen, correspond with these. In some, as in a female skull in the College Museum, the superior incisors are placed as obliquely as in the Negro; but none have so low a forehead and vertex as some of that race.

The arrangement of skulls under the five general forms just described is, in a great measure arbitrary. It must not, therefore, be taken in a strict sense: we must not expect to find all the individuals, comprised under each of these varieties, decisively distinguished by the assigned characters from all others. In the endless diversity of individual forms, many instances are met with, in each variety, of organizations approaching to those of the others; so that among many Europeans and Negroes we might select skulls in which it would be difficult to determine the predominant character. The two intermediate forms between the Caucasian middle, and the Ethiopian and Mongolian extremes, complete the series of gradations. Of the numerous tribes or nations in each division, some come nearer to one and some to the other of the two immediately adjoining varieties.

We must therefore conclude, that the diversities of features and of skulls are not sufficient to authorize us in assigning the different races of mankind in which they occur to species originally different. This conclusion will be strengthened by the analogies of natural history. The differences between human crania are not more considerable, nor even so remarkable, as some variations which occur in animals confessedly of the same species. The head of the wild boar is widely different from that of the domestic pig. The different breeds of horses and dogs are distinguished by the most striking dissimilarities in the skull; in which view, the Neapolitan and Hungarian horses may be contrasted. The very singular form of the skull in the Paduan fowl is a more remarkable deviation from the natural structure than any variation which occurs in the human head.

For the variety of features occurring among the different races of mankind, no natural cause is sufficiently uniform in its operation to afford a satisfactory explanation. It has indeed been asserted by high authority, that climate has a gradual influence in assimilating the countenance of tribes who have left their original abode, to the cast of features prevalent in the country in which they have taken up their habitations. All observation, however, proves the reverse of this. Specific differences prevail over too large an extent of the globe, and under too great a variety of climate to be much under the influence of physical causes. But we have evidence more to the point, in the fact that colonies of the different races have, for centuries, preserved their characteristic features, among a people forming a perfect contrast to them in almost every respect.

Chapter V. treats of *Varieties in Figure, Proportions, Strength, &c.*—We have little room for quotations from this chapter, altho' the subject is far from being an uninteresting one. It is a curious circumstance, and one we think not generally known, that, *cœteris paribus*, individuals of the Caucasian variety are more robust in their form, particularly about their loins, than any other of the families of mankind,—the dimensions of the European being greater than that of the negro in this respect, in the proportion of 1-4 or 1-3. The following quotation shews some:

In order to procure some exact comparative results on this point, Peron took with him on his voyage an instrument called a dynamometre, so constructed, as to indicate, on a dial-plate, the relative force of individuals submitted to experiment. He directed his attention to the strength of the arms and of the loins, making trial with several individuals of each kind; viz. twelve natives of Van Diemen's Land, seventeen of New Holland, fifty-six of the island of Timor, seventeen Frenchmen belonging to the expedition, and fourteen Englishmen in the colony of New South Wales. The following numbers express the mean result in each case; but the details are all given in a tabular form in the original.

| | STRENGTH | |
|---|---|---|
| | of the Arms. Kilogrammes | of the Loins. Myriagrammes. |
| 1. Van Diemen's Land - - - - - - | 50.6 | |
| 2. New Holland - - - - - - - - | 50.8 | 10.2 |
| 3. Timor - - - - - - - - - - - | 58.7 | 11.6 |
| 4. French - - - - - - - - - - | 69.2 | 15.2 |
| 5. English - - - - - - - - - | 71.4 | 16.3 |

The highest numbers in the first and second class were respectively 60 and 62; the lowest in the English trial, 63, and the highest 83, for the strength of the arms. In the power of the loins, the highest among the New Hollanders was 13, the lowest of English 12.7, and the highest 21.3.

It was also remarked in a former part of this work, that one of the distinctions between man and the monkey tribes, was to be found in the comparative length of the ulna. Something like this difference is also found between the European and the other varieties of the human race. From the admeasurement of a number of European skeletons, the humerus was found to exceed the ulna from 2 1-2 to 4 inches, while in the other races the difference is from 1 1-2 to 2 1-4 inches. Upon the account of some other differences, more obvious indeed and curious, but less important in their relations to the present subject, we shall not dwell.

Chapter VI. *On Differences of Stature; Origin and Transmission of Varieties in Form.*—Very considerable differences of stature, both as individual varieties, and as peculiarities of certain tribes, are to be met with. The standard stature in the temperate climates of Europe and America may be considered as between five and six feet. Many well authenticated cases are recorded of individuals falling as low as three feet, on the one hand; and again rising as high as eight feet, and even eight and a half, on the other. But of those who exceed the natural standard, it is seldom observed that their corporeal powers bear any proportion to their size; or that healthy constitutions and good proportions are found among dwarfs.

Beside these individual differences, tribes are found among all the five varieties of the human race, conspicuous for their size and strength, and others remarkable for their small stature and inferior muscular power. External influences are observed to have little effect upon the stature; these varieties not being connected with any peculiarity of temperature, climate, situation, or mode of life. The most remarkable deviations above the common standard, are to be found in the Patagonians of South America and the Kaffers of Africa—most of these tribes reaching to the height of six feet, many to six and a half, and a few excelling seven. It is not a little remarkable that the latter, the Kaffers, are contiguous to the Hottentots, the smallest of all the African race. To the general rule that climate exerts little influence over the human stature, are to be excepted the inhabitants of high northern latitudes,—the Laplanders, Samoides, Ostiacks, Greenlanders, Esquimaux, &c., all of whom are remarkably short in their stature, and very ill-proportioned, although muscular and robust.

In accounting for the diversities of the human race, Mr. L., after alluding to the numberless varieties in form, size, color, and other external qualities, which are found among the domestic animals, varieties much greater than any occurring among mankind, makes the following remarks:

In endeavoring to account for the diversities of features, proportions, general form, stature, and the other particulars mentioned in the three preceding chapters, I must repeat an observation already made and exemplified in speaking of color; namely that the law of resemblance between parents and offspring, which preserves species, and maintains uniformity in the living part of creation, suffers occasional and rare exceptions; that, under certain circumstances, an offspring is produced with new properties, different from those of the progenitors; and that the most powerful of these causes is that artificial mode of life which we call the state of domestication.

Native or congenital peculiarities of form, like those of color, are transmitted by generation. Hence we see a general similitude in persons of the same blood; and can distinguish one brother by his resemblance to another, or know a son by his likeness to the father or mother, or even to the grandfather or grandmother. All the individuals of some families are characterized by particular lines of countenance: and we frequently observe a peculiar feature continued in a family for many generations. The thick lip introduced into the Imperial house of Austria, by the marriage of the Emperor Maximilian with Mary of Burgundy, is visible in their descendants to this day, after a lapse of three centuries. Haller observes, that his own family had been distinguished by tallness of stature for three generations, without excepting one out of numerous grandsons descended from one grandfather.

Individuals are occasionally produced with supernumerary members on the hands or feet, or on both; and from these, whether males or females, the organic peculiarity frequently passes to their children.—This does not constantly happen, because they intermarry with persons of the ordinary form; but if the six-fingered and six-toed could be matched together, and the breed could be preserved pure by excluding all who had not these additional members, there is no doubt that a permanent race might be formed constantly possessing this number of fingers and toes.

Another remarkable example of the occurrence of a singular organic peculiarity, and of its hereditary transmission, is afforded by the English family of porcupine men, who have derived that name from the greater part of the body being covered by hard dark colored excrescences of a horny nature. The whole surface, excepting the head and face, the palms and soles, is occupied by this unnatural kind of integument. The first account of this family is found in the *Philosophical Transactions*, No. 423, and consists of the description of a boy named Edward Lambert, fourteen years old, born in Suffolk, and exhibited to the Royal Society in 1731, by Mr. Machin, one of the secretaries.

They are shed annually, in the autumn or winter, and succeeded by a fresh growth, which at first are of a paler brown. "He has had the small pox, and been twice salivated, in hopes of getting rid of this disagreeable covering; during which disorders the warts came off, and his skin appeared white and smooth, like that of other people; but on his recovery it soon became as it was before. His health at other times has been very good during his whole life." "He has had six children, all with the same rugged covering as himself; the first appearance whereof in them, as well as in him, came on in about nine weeks after the birth. Only one of them is living, a very pretty boy, eight years of age, whom I saw and examined with his father, and who is exactly in the same condition."

Two brothers, John Lambert, aged twenty-two, and Richard, aged fourteen, who must have been grandsons of the original porcupine man, Edward Lambert, were shown in Germany, and had the cutaneous incrustation already described. A minute account of them was published by Dr. W. G. Tilesius, who mentions that the wife of the elder, at the time he saw him, was in England, pregnant.

This resemblance of offspring to parents, in native peculiarities of structure, prevails so extensively, that those minute and in many cases imperceptible, differences of organization or vital properties, which render men disposed to particular diseases, are conveyed from father to son for age after age. This is matter of common notoriety with respect to scrofula, consumption, gout, rheumatism, insanity, and other affections of the head. There is more doubt in some other cases, as hare-lip, squinting, club-foot, hernia, aneurism, cataract, fatuity, &c.; of which, however, there are many well authenticated examples. There is an hereditary blindness in a family in North America, which has always affected some individuals for the last hundred years. I have attended, at different times, for complaints of the urinary organs, a gentleman, whose father and grandfather died of stone.

In small and secluded communities, where marriages take place within what we may regard only as a more extensive family, hereditary varieties are blended, and produce one form, which prevails through the whole circle. The operation of this principle may be clearly perceived in several small districts: it will act with more efficacy, and, consequently, be more discernable in larger collections of men, where differences of manners, religion, and language, and mutual animosities, forbid all intermarriages with surrounding people. In the course of time the individual peculiarities are lost, and a national characteristic countenance or form is established, which, if the restrictions of intercourse are rigidly adhered to, is constantly more and more strengthened. The ancient Germans, according to the description of Tacitus, were such a people; and his short, but expressive sketch of their character, most aptly confirms the preceding view: "Ipse eorum opinionibus accedo, qui Germaniæ populos nullis aliarum nationum connubiis infectos, propriam et sinceram et tantum sui similem gentem exitisse arbitrantur. Unde habitus quoque corporum, quanquam in tanto hominum numero, idem omnibus; truces et cærulei oculi, rutilæ comæ, magna corpora." *De Morib. Germ.*

The Gipsies afford another example of a people spread over all Europe for the last four centuries, and nearly confined in marriages, by their peculiar way of life, to their own tribe. In Transylvania, where there is a great number of them, and the race remains pure, their features can consequently be more accurately observed: in every country and climate, however, which they have inhabited, they preserve their distinctive character so perfectly, that they are recognized at a glance, and cannot be confounded with the natives. But, above all, the Jews exhibit the most striking instance of a peculiar national countenance, so strongly marked in almost every individual, that persons the least used to physiognomical observations detect it instantly, yet not easily understood or described. Religion has in this case most successfully exerted its power in preventing communion with other races; and this exclusion of intercourse with all others has preserved the Jewish countenance so completely in every soil and climate of the globe, that a miracle has been thought necessary to account for the appearance.

In what other way can we explain the difference between the English and Scotch? Would it be more reasonable to suppose that they descended from different stocks; or to ascribe the high cheek bones of the latter to the soil or climate?

As, on the one hand, a particular form may be perpetuated by confining the intercourse of the sexes to individuals in whom it exists: so, again, it may be changed by introducing into the breed those remarkable for any other quality. Connexions in marriage will generally be formed on the idea of human beauty in any country; an influence, this, which will gradually approximate the countenance towards one common standard. If men, in the affair of marriage, were as much under management as some animals are in the exercise of their generative functions, an absolute ruler might accomplish, in his dominions, almost any idea of the human form.

The great and noble have generally had it more in their power than others to select the beauty of nations in marriage: and thus, while, without system or design, they gratified merely their own taste, they have distinguished their order, as much by elegant proportions of person, and beautiful features, as by its prerogatives in society. "The same superiority," says Cook, "which is observable in the crees or nobles in all the other islands, is found here (Sandwich Islands.) Those, whom we saw, were, without exception, perfectly well-formed; whereas, the lower sort, besides their general inferiority, are subject to all the variety of make and figure that is seen in the populace of other countries.

In no instances, perhaps, has the personal beauty of a people been more improved, by introducing handsome individuals to breed from, than in the Persians, of whom the nobility have, by this means, completely succeeded in washing out the stain of their Mongolian origin. "That the blood of the Persians," says Chardin, "is naturally gross, appears from the Guebres, who are a remnant of the ancient Persians, and are an ugly, ill-made, rough skinned people. This is also apparent from the inhabitants of the Provinces in the neighborhood of India, who are nearly as clumsy and deformed as the Guebres, because they never formed alliances with any other tribes. But, in the other parts of the kingdom, the Persian blood is now highly refined by frequent intermixtures with the Georgians and Circassians, two nations which surpass all the world in personal beauty. There is hardly a man of rank in Persia who is not born of a Georgian or Circassian mother; and even the King himself is commonly sprung on the female side, from one or other of these countries. As it is long since this mixture commenced, the Persian women have become very handsome and beautiful, though they do not rival the ladies of Georgia. The men are generally tall and erect, their complexion is ruddy and vigorous, and they have a graceful air and an engaging deportment. The mildness of the climate, joined to their temperance in living, has a great influence in improving their personal beauty. This quality they inherit not from their ancestors: for without the mixture mentioned above, the men of rank in Persia, who are descendants of the Tartars, would be extremely ugly and deformed."

There is no one of the varieties above enumerated, which does not exist in a still greater degree in animals confessedly of the same species. What differences in the figure and proportion of parts in the various breeds of horses—in the Arabian, the Barb, and the German! How striking the contrast between the long-legged cattle of the Cape of Good Hope and the short-legged of England! The same difference is observed in swine. The cattle have no horns in some breeds of England and Ireland: in Sicily, on the contrary, they have very large ones. A breed of sheep, with an extraordinary number of horns, as three, four, or five, (ovis plycerata,) occurs in some northern countries; as, for instance, in Iceland, and is accounted a mere variety. The Cretan breed of the same animal (ovis strepsiceros) has long, large, and twisted horns. We may also point out the solid-ungular swine, with undivided hoof, as well as others with three divisions of that part; the five-toed fowl (gallus pentadactylus;) the fat-rumped sheep of Tartary and Thibet; and broad-tailed breed of the Cape, in which the tail grows so large that it is placed on a board supported by wheels, for the convenience of the animal; and the rumpless fowl (gallus ecaudatus) of America, and particularly Virginia, which has undoubtedly descended from the English breed.

The hereditary transmission of physical and moral qualities, so well understood and familiarly acted on in the domestic animals, is equally true of man. A superior breed of human beings could only be produced by selections and exclusions similar to those so successfully employed in rearing our more valuable animals. Yet, in the human species, where the object is of such consequence, the principle is almost entirely overlooked. Hence all the native deformities of mind and body, which spring up so plentifully in our artificial mode of life, are handed down to posterity, and tend by their multiplication and extension to degrade the race. Consequently, the mass of the population in our large cities will not bear a comparison with that of savage nations, in which if imperfect or deformed individuals should survive the hardships of their first rearing, they are prevented, by the kind of aversion they inspire, from propagating their deformities. The Hottentots have become almost proverbial for ugliness; and one of their tribes, the Bosjesmen, are plainly marked, by an acute and intelligent traveller, "among the ugliest of human beings." The numerous sketches of Bosjesmen and Hottentots, taken by Mr. S. Daniel, have been very kindly and politely shown to me by his brother, Mr. W. Daniel. In form, variety, and expression of countenance, they are not at all inferior to our cockneys; while in animation, in beauty, symmetry, and strength of body, in ease and elegance of attitude, they are infinitely superior.

This inattention to breed is not, however, of so much consequence in the people, as in the rulers; in those to whom the destinies of nations are intrusted; on whose qualities and actions depend the present and future happiness of millions. Here, unfortunately, the evil is at its height: laws, customs, prejudices, pride, bigotry, confine them to intermarriages with each other; and thus degradation of race is added to all the pernicious influences inseparable from such exalted stations. What result should we expect, if a breeder of horses or dogs were restricted in his choice to some ten or twenty families taken at random? if he could not step out of this little circle, to select finely-formed or high-spirited individuals? How long a time would elapse before the fatal effects of this in-breeding would be conspicuous in the degeneracy of the descendants? The strongest illustration of these principles will be found in the present state of many royal houses in Europe; the evil must be progressive, if the same course of proceeding be continued.

I shall cite a single example to prove what will, to most persons, seem unnecessary; namely, that mental defects are propagated, as well as corporeal. "We know," says Haller, "a very remarkable instance of two noble females, who got husbands on account of their wealth, although they were nearly idiots, and from whom this mental defect has extended for a century into several families; so that some of all their descendants still continue idiots in the fourth and even in the fifth generation."

*Chapters VII. and VIII.* are taken up with an account of the *Differences of Language*, and of *Moral and Intellectual Qualities.* On the subject of language, we have room for the following extract only:

One of the most curious points in the subject of language is the continued existence in a large portion of Asia, very anciently civilized, and considerably advanced at least in the useful arts, of simply monosyllabic languages. Their words are merely radical sounds of one syllable, not admitting of inflection or composition, so that all modifications and accessory ideas must be either overlooked or imperfectly expressed by tedious and awkward circumlocution. Such are the languages of Thibet, the contiguous immense empire of China, and the neighboring countries of Ava, Pegu, Siam, Tungquin, and Cochin-china. "These extensive regions, and these only in the whole world, betray in their present language all the imperfection of the first attempts at speech. As the earliest efforts of the infant are merely sounds of one syllable, so the first adult children of Nature stammered out their meaning in the same way: the people of Thibet, China, and the neighboring southern countries, go on speaking as they learned some thousands of years ago, in the cradle of the species. There is no separation of ideas into certain classes, such as produce the distinction of the parts of speech in more perfectly-formed languages.—One and the same sound signifies *joyful, joy*, and *to rejoice;* and that through all persons, numbers, and tenses. No attempt is made, by affixing sounds expressive of relations or accessory notions to the simple monosyllabic root, to give richness, clearness, and harmony to the poor language. On the contrary, the mere radical ideas are set down together, and the hearer must guess at the connecting links. As there are no inflections, the cases and numbers are either not noted, or they are marked, under urgent circumstances, by circumlocution. They form plurals as children do, either by repetition, as *tree, tree,* or by adding the words *much* or *other;* as *tree much, tree other. I much,* or *I other*, means *we. Be Heaven I other Father who*, is the mode of expressing "Our Father which art in heaven!" "That languages of such poverty, which merely place together the most essential ideas without connecting them, must open a wide field for ambiguity and obscurity in civil life, and be totally inapplicable to the purposes of science, is immediately apparent. Hence the people who speak them must ever remain children in understanding. However the Chinese may exert themselves, so long as they are impeded by this imperfect language, they must be unable to appropriate to themselves the sciences and arts of Europe."

It is a matter of surprise that this peculiarity of language does not extend to many of the neighboring nations, who very closely resemble the Chinese in external conformations, as well as in moral and social habits and religious institutions. The Japanese have a well-formed polysyllabic language, without any resemblance to that of the Chinese. The languages of the American tribes of Indians form a curious contrast to that of the Chinese, in the length of their words, and in the capability of inflection and composition.

The causes of the diversities of language are very imperfectly understood. It is easy to conceive that the various degrees of natural capacity, and of intellectual and social improvement, will very materially influence both the structure and the copiousness of language. Language also will be not less influenced in its structure, copiousness, and precision, by the arts of writing and printing. Of the extent of this influence, an imperfect idea may be formed by comparing the powers of language possessed by an individual of a highly cultivated mind, with one of the same nation destitute of the advantages of intellectual improvement. Even this comparison, however, will convey a very imperfect idea of the improvement which language derives from writing, for the language of the most untutored individual, must partake, to a certain extent, of the intellectual improvements of the society in which he lives. Such is the influence of a written language; but hieroglyphic writing, like that of the Chinese, can tend but very little to the improvement of the language of a country. By affording a species of intellectual labor to men of leisure, it may tend, in some degree, to rescue those who direct public opinion from the degrading influence of sensual indulgence, and thus lead to the prosecution of arts which may give some dignity and refinement to society at large; but still, a people who have no better method of preserving and transmitting the discoveries of genius, must always remain children in science, and stationary in improvement. The wisdom and learning of their sages will be wasted upon trifles—upon the accumulation of proverbs and maxims; and their artists, unaided by complicated machinery, will literally earn their bread by the sweat of their brow.

On the subject of the moral and intellectual qualities, Mr. L. is disposed to attribute to the Caucasian variety the most unqualified superiority. If history could decide the question, their claims would indeed be indisputable. This variety of mankind alone have made great advances in learning and civilization; and among them only, have attempts been made, on a large scale, to subject the elements to the dominion of man, or to establish the government of reason, intellect, and religion, over passion and brute force.

That there is a general intellectual superiority in the white races, all must admit; and when we know that the white races have always enjoyed a degree of civilization far superior to that of the other varieties of mankind, much of this difference may be attributed to the operation of a very general and familiar law. The law to which we refer is this, that intellectual qualities, as well as physical peculiarities, are hereditary. Now in the same way that national ideas of beauty will have an influence in regulating the physiognomy of a people, will the estimate they put upon intellect, have a gradual though imperceptible influence in elevating or depressing the intellectual character. Admitting that the strength of the intellect depends upon the development of the brain, if it were merely a matter of national taste that high foreheads were more beautiful than depressed and retreating ones, this simple fact, upon the principles of Mr. L. himself, would have its influence in affecting the conformation of the head, and consequently of improving the intellect. In addition to this, among all savage nations, in proportion to their degradation, strength and agility are prized above wisdom and intelligence; and where polygamy prevails, and where individual character and the sustenance of families depends entirely upon the exertion of these corporeal qualities, their influence would be far greater upon the character, than could possibly result from national ideas of personal beauty upon the physiognomy among a civilized people.

Much of the weight of Mr. L.'s reasoning upon this subject depends upon the assumption of the fact that the different nations of the world were once equally barbarian, and that language, science, and religion, are human inventions—an assumption purely gratuitous, and in opposition to which, the strongest arguments, drawn from history, and observation, can be adduced. There is indeed every reason to believe, that nations, destitute of letters, and placed below a certain grade of civilization, will continue to retrograde in every respect, nntil they become the most worthless of savages. If man has derived his existence from a superior being, it requires no great stretch of credulity to suppose, that his Creator would bestow upon him all the gifts necessary to the enjoyment of that social condition for which he is evidently intended, and that he should give him, among the rest, a knowledge of himself, and a medium of social intercourse. In other words, if God intended to people the earth with intelligent creatures, it is a fair presumption that he would create the parents of the race in full perfection, that he would make them—not children, but men, in knowledge and virtue, as well as in stature.

It is much more easy to conceive that different colonies setting out from the patriarchal government would carry with them varying degrees of knowledge and religion, and that some, from adverse circumstances, would lose the little they possessed, while others, placed in situations favorable to improvement, might make great advances in civilization and refinement; than to believe that naked savages, associated by accident or for mutual defence, would invent language, religion, and letters. Improvement in science or the arts, proceeds in a regular gradation, each series resting upon the one that precedes it. The tendency of mankind at large is, to tread with faithful servility in the steps of their ancestors. Inventive genius is rare, and its successful exertion presupposes on the part of the individual, education, (the acquisition of which, without some kind of oral communication, is inconceivable,) and, on the part of the society, encouragement and assistance. It is easy to believe, therefore, that in a state of society below a certain grade of intellectual culture, circumstances may arise, under which, genius, from want of encouragement, will ever remain inactive, or its solitary labors, disregarded or unappreciated, fail in making any useful impression upon society.

It is further to be recollected, that, in making a cursory comparison between civilized and savage nations, impressed with the striking contrast which their respective social condition exhibits, we are apt to attribute to the former a much greater intellectual superiority than individual observation will justify.

The refinement of a nation depends very much upon accidental circumstances—upon its prosperity, its language, its literature, and, above all, its religion. In the best informed communities, we find wide-spread ignorance, error, prejudice, superstition and bigotry, and large bodies of men adopting implicitly the opinions of their superiors, and dependent on them for all that they enjoy of education or civilization.

If "the three religions which exhibit the only worthy views the Deity," are mere human inventions, to be set down to the credit of the Caucasian variety, (not a very modest assumption, by the way,) this highly gifted race can derive very little honor from their discovery, since the first was confined for centuries to a small nation, so insensible to its value as to be continually running into the grossest superstitions of their neighbors; the principles of the second, the most sublime of them all, were discovered by an obscure individual, and made their way into notice through the aid of the lower orders of society, amidst the persecution and philosophy of the times; while the last of the three, a mere excrescence from the two former, was established by the sword of a set of banditti.

The same remark may be applied to the art of writing. Notwithstanding the fact, that many nations of the Caucasian variety have been destitute of learning for centuries, authentic history gives no account of a Cadmus, except in the instance of an uneducated Cherokee, who has, within a few years, invented a syllabic alphabet adapted to his native language. All history in the mean time discountenances the presumption that the condition of any tribe of savages has been materially improved in any other way than by the importation of the knowledge and arts of their more civilized neighbors.

The American savages are almost universally distinguished for their bravery and love of liberty. Men truly great have also appeared among them, but adverse circumstances have controlled their destiny, and rendered abortive their labors for the improvement of their country. Nor is this surprising among savages, when we consider the reception of reformers among much more enlightened people,—of Socrates, Wickliffe,

Huss, Galileo, and Vesalius; the annihilation of the Waldenses, the persecution of Luther, and the expulsion of the Huguenots; when we recollect that Milton was compelled to appeal to posterity in behalf of Paradise Lost, and that Newton's theory of gravitation was long a subject of ridicule among the learned; and when we have been compelled to witness, in our own enlightened age, those whose discoveries have since revolutionized the commerce and the manufactures of the world, living upon charity, the very men who fed them ashamed of their society, and dying at last of chagrin and mortification, without being permitted to witness the result of their labors.

When the author speaks of the comparative humanity of the white races to their conquered enemies, he forgets that war itself, carried on for the purpose of ambition or aggrandisement, is the height of inhumanity, and overlooks the more palpable brutality of the Roman gladiatorial exhibitions justified by the philosophic Cicero, the cruelties of the Triumviri and the Emperors, the Inquisition, the massacre of St. Bartholomew, the war between Spain and the Netherlands, and the ravages of the former power in the New World, the French revolution, with the unnumbered instances of violence and oppression which deform the pages of history. Tecumseh and King Philip are represented, as far as their education and circumstances permitted, to have been generous and humane; and if Marlborough, Eugene and Napoleon do seem to have possessed more of the milk of human kindness, it proves very little more than that the discipline of the camp had not eradicated from their minds a decent regard for the opinions of mankind, nor the practice of war, by entirely closing the heart against the claims of humanity, imparted a disposition to aggravate, unnecessarily, the suffering which their ambition was calculated to produce.

The question of the possibility of civilizing the colored races of mankind has not yet been made the subject of fair experiment. In accomplishing this object, the friends of humanity have difficulties of the utmost magnitude to encounter—to supplant their religion, to enlighten their minds, to substitute industry for the love of ease or the fascinating pleasures of the chase, to counteract the pernicious influence of vicious white men, and to remove the prejudices which must prevail against men who have heretofore come to their abode only to cheat and oppress them.

If British commerce with India had been based upon the broad foundation of justice and mutual interest, instead of conquest and oppression, and if the European merchant had enriched Africa, by encouraging the inexhaustible resources of her luxuriant climate, instead of draining her vitals and debasing her morals, by the sale of her children, the social aspect of those countries would at this time have been very different.—The experiment is now making under circumstances calculated to give the question a definitive solution, and with prospects of final and complete success. This result cannot fail of being attended with the most beneficial consequences in drawing closer the ties by which the different nations of the earth are connected together; for although the inferiority of our brethren is far from affording a justification for depriving them of their rights, yet it is a fact that this absurd plea is, at all times, an important item in the defence of cruelty and oppression.

